# Association between abnormalities on chest computed tomography and pulmonary function in patients with respiratory symptoms at 12 months after COVID-19

**DOI:** 10.1590/0100-3984.2025.0043

**Published:** 2025-11-07

**Authors:** Luciano Folador, Vicente Bohrer Brentano, Ravena Maya Cardoso da Silva, Igor Gorski Benedetto, Marcelo Basso Gazzana, Danilo Cortozi Berton, Tiago Severo Garcia

**Affiliations:** 1 Hospital de Clínicas de Porto Alegre (HCPA), Porto Alegre, RS, Brazil.; 2 Faculdade de Medicina da Universidade Federal do Rio Grande do Sul (UFRGS), Porto Alegre, RS, Brazil.

**Keywords:** Post-acute COVID-19 syndrome, Tomography, X-ray computed, Pulmonary fibrosis, SARS-CoV-2, COVID-19/complications, Síndrome pós-COVID-19 aguda, Tomografia computadorizada por raios X, Fibrose pulmonar, SARS-CoV-2, COVID-19/complicações

## Abstract

**Objective:**

To assess the prevalence and type of residual lung abnormalities on chest computed tomography (CT) and pulmonary function testing (PFT) variables in patients with respiratory symptoms related to post-COVID-19 condition at 12 months of follow-up, and to analyze associations between CT findings and PFT parameters.

**Materials and Methods:**

The CT findings were evaluated by two radiologists, who calculated semiquantitative CT scores. The PFTs included spirometry, plethysmography, and the diffusing capacity of the lung for carbon monoxide.

**Results:**

Thirty-seven patients were included in the study. On CT scans of the chest acquired at 12 months of follow-up, 78.3% of the patients exhibited residual abnormalities, including reticular opacities, in 75.7%; traction bronchiectasis/bronchiolectasis, in 43.2%; and fibrosis-like findings, in 43.2%. The mean overall CT score was 9.30 ± 2.59. Patients with fibrosis-like findings had significantly lower total lung capacity (68.6% vs. 80.6% of the predicted value; *p* = 0.018). A moderate negative correlation was found between the overall CT score and total lung capacity (rs = −0.49; *p* = 0.003).

**Conclusion:**

It seems that a significant proportion of patients with respiratory symptoms related to post–COVID-19 condition demonstrate residual lung abnormalities on chest CT at 12 months of follow-up, with a substantial prevalence of fibrosis-like findings. Such abnormalities are associated with restrictive lung disease.

## Abstract (pt)

**Objetivo:**

Avaliar a prevalência e o tipo de anormalidades pulmonares residuais em tomografias computadorizadas (TCs) de tórax e provas de função pulmonar (PFP) de pacientes sintomáticos respiratórios pós-COVID-19 após 12 meses e analisar as associações dos achados de imagem e PFP.

**Materiais e Métodos:**

Os achados tomográficos foram avaliados por dois radiologistas e foi calculado um escore semiquantitativo na TC. As PFPs incluíram espirometria, pletismografia e a capacidade de difusão pulmonar para monóxido de carbono.

**Resultados:**

Trinta e sete pacientes foram incluídos no estudo. Aos 12 meses, 78,3% dos pacientes apresentaram anormalidades residuais na TC de tórax, incluindo opacidades reticulares (75,7%), bronquiectasias/bronquiolectasias de tração (43,2%) e achados semelhantes a fibrose (43,2%). O escore global médio de TC foi de 9,30 ± 2,59. Pacientes com achados semelhantes a fibróticos apresentaram capacidade pulmonar total prevista menor (68,6% vs. 80,6%; *p* = 0,018). Foi encontrada uma correlação negativa moderada entre o escore global da TC e a capacidade pulmonar total prevista (rs = –0,49; *p* = 0,003).

**Conclusão:**

Uma proporção significativa de pacientes sintomáticos respiratórios pós-COVID demonstrou anormalidades pulmonares residuais na TC de tórax em 12 meses, com prevalência substancial de achados semelhantes a fibrose. Esses achados foram associados a distúrbios ventilatórios restritivos.

## INTRODUCTION

Severe acute respiratory syndrome coronavirus 2 (SARS-CoV-2) has infected over 750 million people worldwide^(1)^. As defined by the UK National Institute for Health and Care Excellence^(2)^, there are three clinical stages of SARS-CoV-2 infection: acute (the first 4 weeks after diagnosis); ongoing symptomatic (4–12 weeks after diagnosis); and post-COVID-19 syndrome (> 3 months after diagnosis). Research suggests that 10–20% of patients with COVID-19 develop prolonged symptoms associated with post-COVID-19 condition^(3)^. Common residual symptoms in this group of patients are fatigue and dyspnea; those in whom the acute phase of COVID-19 was severe or critical are at higher risk of experiencing long-term post-COVID-19 symptoms.

Recent reviews and meta-analyses of chest computed tomography (CT) findings at 6–12 months after COVID-19 have reported a wide range of prevalence rates for post-COVID-19 abnormalities on CT^(4^,^5)^. In addition, a substantial number of COVID-19 survivors have been shown to exhibit chronic abnormalities on pulmonary function tests (PFTs), with impaired diffusing capacity of the lung for carbon monoxide (DL_CO_) and a restrictive pattern of lung function at 12 months after COVID-19 pneumonia^(6)^.

The aim of the present study was to assess the prevalence and type of residual lung abnormalities on chest CT and PFT variables in patients with respiratory symptoms related to post-COVID-19 condition (long COVID) at 12 months of follow-up. We also aimed to analyze the association between the CT findings and PFT parameters.

## MATERIALS AND METHODS

### Patients

The subjects in this study were nested within a cohort of adult survivors of severe COVID-19 who were followed at the pulmonology clinic of a tertiary care teaching hospital^(7)^. We enrolled patients ≥ 18 years old who had been hospitalized for severe COVID-19 pneumonia between March 31, 2020, and November 23, 2022, and followed for at least 12 months after discharge. Laboratory confirmation of SARS-CoV-2 infection was defined as a positive reverse transcription-polymerase chain reaction result from a nasal swab sample. Severe COVID-19 was defined as fever or suspected lower respiratory tract infection plus one of the following criteria^(8)^: respiratory rate > 30 breaths/min; severe respiratory distress or oxygen saturation ≤ 93% on room air; and pulmonary infiltrates > 50% on chest imaging in the first 24–48 h after hospital admission. During each follow-up visit (at 3 and 12 months after diagnosis), the study subjects underwent full PFTs and completed questionnaires to evaluate health-related quality of life and respiratory symptoms.

We selected symptomatic patients with post-COVID-19 condition, defined as the persistence of respiratory symptoms (dyspnea, cough, and sputum production) for > 3 months after acute COVID-19, who underwent chest CT at 12 months of follow-up. Subjects with non-respiratory symptoms were not included in our analysis. Patients with active respiratory tract infection at the time of the CT were excluded, as were those who were currently undergoing cancer treatment, those with extensive pulmonary scarring from previous granulomatous infections, and those with any clinical condition that would preclude the performance of the study procedures. Data regarding outcome measures were obtained from a cross-sectional analysis of the data.

The study was approved by the local research ethics committee (Reference no. 2020-0169) and was performed in accordance with the Declaration of Helsinki. All participating patients gave written informed consent. The study protocol was registered at ClinicalTrials.gov (Identifier: NCT04410107). There are no conflicts of interest to declare.

### Procedures

The modified Medical Research Council dyspnea scale was employed to grade dyspnea during activities of daily living^(9)^, with scores ranging from 0 (absence of dyspnea during strenuous exercise) to 4 (too breathless to leave the house or breathless while dressing or undressing). Cough and sputum production were assessed with an adapted translation of the American Thoracic Society respiratory symptoms questionnaire^(10)^.

Spirometry, body plethysmography, and single-breath DL_CO_ measurement were performed in accordance with the American Thoracic Society/European Respiratory Society standards^(11^–^13)^, with the use of an automated system (MasterScreen PFT; CareFusion, Yorba Linda, CA, USA). An obstructive pattern of lung function, as evidenced by a reduction in the forced expiratory volume in one second/forced vital capacity (FEV_1_/FVC) ratio after bronchodilator administration, was characterized by measurements below the lower limit of normal (i.e., below the −1.645 z-score), as were a restrictive pattern of lung function, reduced total lung capacity (TLC, expressed as a percentage of the predicted value), and reduced DL_CO_^(14)^.

Images were obtained in an eight-row multidetector CT scanner (BrightSpeed Edge; GE Medical Systems, Milwaukee, WI, USA), a 16-row multidetector CT scanner (Brilliance 16; Philips Healthcare, Best, The Netherlands), or a 64-row multidetector CT scanner (Aquilion 64; Toshiba Medical Systems, Tokyo, Japan), with patients in the supine position and at full inspiration. All scans were volumetric acquisitions (slice thickness: 1.0–2.0 mm) and were reconstructed with a high-spatial-frequency algorithm. Images were stored and analyzed with a picture archiving and communication system (IMPAX, version 6.6.1.3525; Agfa HealthCare, Mortsel, Belgium). When used, iodinated nonionic intravenous contrast medium was injected into a peripheral vein at a dose of 1–2 mL/kg of body weight.

**Figure 1:**
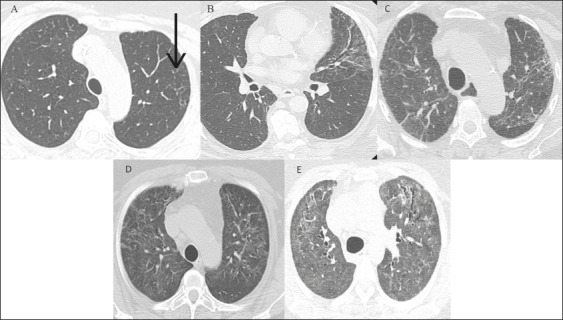
Semiquantitative CT scoring system. **A:** Involvement of < 5% (arrow). **B:** Involvement of 5–25%. **C:** Involvement of 26–49%. **D:** Involvement of 50–75%. **E:** Involvement > 75%.

All of the images were evaluated by two thoracic radiologists with 8 and 12 years of experience, respectively, working independently; both were blinded to the clinical data and laboratory test results. Imaging characteristics were described as ground-glass opacities, reticular opacities, parenchymal bands, traction bronchiectasis/bronchiolectasis, and honeycombing, based on the standard glossary for thoracic imaging published by the Fleischner Society^(15)^. Traction bronchiectasis/bronchiolectasis and honeycombing were further classified as fibrosis-like findings because they are associated with parenchymal distortion and irregular bronchopulmonary and pleuropulmonary interfaces^(16)^. A semiquantitative scoring system^(17)^ was used in order to assess the extent of lung involvement on chest CT scans. Each of the five lung lobes was visually scored on a scale of 0 to 5, with 0 indicating no involvement; 1 indicating involvement of < 5%; 2 indicating involvement of 5–25%; 3 indicating involvement of 26–49%; 4 indicating involvement of 50–75%; and 5 indicating involvement of > 75%. The total CT score was the sum of the individual lobar scores, therefore ranging from 0 (no involvement) to 25 (maximum involvement). Discrepancies between the two assessors regarding pulmonary findings were resolved by consensus. Figure 1 exemplifies the semiquantitative CT scoring system. The mean interobserver kappa value for qualitative variables was 0.72 [95% confidence interval (95% CI): 0.63–0.79] and the intraclass correlation coefficient for the extent of lung involvement on chest CT was 0.83 (95% CI: 0.70–0.91).

### Statistical analysis

Statistical analysis was performed with the IBM SPSS Statistics software package, version 20.0 (IBM Corp., Armonk, NY, USA). Categorical variables are presented as frequencies and percentages. Prevalence values are presented with their respective 95% CIs. Quantitative variables, after being evaluated for their symmetry with the Kolmogorov-Smirnov test, are presented as mean and standard deviation (SD) or as median and interquartile range, as appropriate. Categorical variables were assessed with the chi-square test or Fisher’s exact test. Quantitative variables were compared by using the Student’s t-test for independent samples. Correlations between quantitative variables were assessed by calculating Spearman’s rho (r_s_). Values of *p* < 0.05 were considered statistically significant.

## RESULTS

There were 120 patients from the cohort in follow-up. Of those, 52 underwent chest CT at 12 months of follow-up and 42 of those patients were symptomatic. We excluded four patients because of extensive pulmonary scarring from previous granulomatous infections and one patient because of ongoing cancer treatment. The characteristics of the remaining 37 patients are summarized in Table 1. Table 2 details the symptoms and PFT results. The most common symptoms were dyspnea (in 94.6%), cough (in 59.5%), and sputum production (in 29.7%). The overall prevalence of a restrictive pattern of lung function was 63.9%. Reduced DL_CO_ was found in 62.1%. Because none of the patients showed an obstructive pattern of lung function on spirometry (using the lower limit of normal or a fixed FEV_1_/FVC ratio below 0.7), no further evaluation of obstructive defects was performed.

**Table 1:** Demographic, clinical, and symptomatological characteristics of patients with respiratory symptoms at 12 months after COVID-19.

Characteristic	(N = 37)
Age (years), mean ± SD	58.2 ± 11.6
Sex, n (%)	
Female	17 (45.9)
Male	20 (54.1)
Race, n (%)	
White	31 (83.9)
Black	6 (16.2)
Current or former smoker, n (%)	19 (51.4)
Pulmonary comorbidity, n (%)	7 (18.9)
Chronic obstructive pulmonary disease, n (%)	5 (13.5)
Asthma, n (%)	2 (5.4)
Cardiovascular comorbidity, n (%)	4 (10.8)
Hypertension, n (%)	19 (51.4)
Diabetes, n (%)	11 (29.7)
Obesity, n (%)	21 (56.8)
Neurological comorbidity, n (%)	6 (16.2)
COVID-19 complicated by acute respiratory distress	
syndrome, n (%)	26 (70.3)
Intensive Care Unit admission, n (%)	27 (73.0)
Tracheal intubation, n (%)	18 (48.6)
Hospital stay (days), median (IQR)	26 (12-55)

**Table 2:** Symptoms and PFT variables in patients with respiratory symptoms at 12 months after COVID-19.

Variable	(N = 37)
Dyspnea,n (%)	35 (94.6)
mMRC dyspnea scale score, n (%)	
1	9 (25.7)
2	7 (20.0)
3	12 (34.3)
4	7 (20.0)
Cough, n (%)	22 (59.5)
Expectoration, n (%)	11 (29.7)
Spirometry parameters	
FVC (% predicted), mean ± SD	76.1 ± 17.3
FEV_1_ (% predicted), mean ± SD	82.9 ± 18.9
FEV_1_/FVC, mean ± SD	0.80 ± 0.02
FEV_1_/FVC < LLN, n (%)	0 (0.0)
Plethysmography*	
TLC (% predicted)	76.1 ± 15.4
TLC < LLN, n (%)	23 (63.9)
RV (% predicted)	93.2 ± 28.5
RV < LLN, n (%)	3 (8.3)
Diffusing capacity of the lung	
DL_CO_ (% predicted), mean ± SD	70.4 ± 20.5
DL_CO_ < LLN, n (%)	23 (62.1)

mMRC, modified Medical Research Council; LLN, lower limit of normal; RV, residual volume.

* Data available for only 36 of the 37 patients in the sample.

On chest CT (Table 3), we identified isolated ground-glass opacities in seven patients (18.9%), reticular opacities in 28 (75.7%), parenchymal bands in 11 (29.7%), traction bronchiectasis/bronchiolectasis in 16 (43.2%), and honeycombing in 9 (24.3%). Fibrosis-like findings were present in 16 (43.2%) of the patients. The mean overall CT score for the extent of lung involvement was 9.30 ± 2.59.

**Table 3:** Prevalence of chest CT findings in patients with respiratory symptoms at 12 months after COVID-19.

Finding	(N = 37)
Any residual abnormality, n (%)	29 (78.3)
Ground-glass opacities, n (%)	7 (18.9)
Reticular opacities, n (%)	28 (75.7)
Parenchymal bands, n (%)	11 (29.7)
Traction bronchiectasis/bronchiolectasis, n (%)	16 (43.2)
Honeycombing, n (%)	9 (24.3)
Finding(s) indicative of fibrosis, n (%)	16 (43.2)
Overall CT score, mean ± SD	9.30 ± 2.59

**Figure 2:**
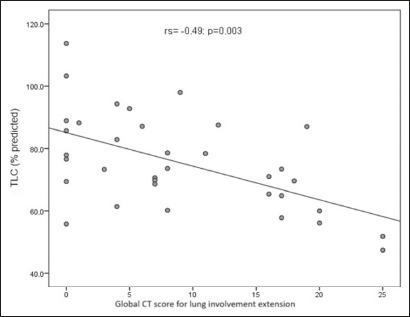
Correlation between the overall CT score for the extent of lung involvement and the TLC in patients with respiratory symptoms at 12 months after COVID-19 (N = 37).

The CT findings and PFT parameters at follow-up are compared in Table 4. The TLC was significantly lower in the patients with fibrosis-like findings than in those without (68.6% vs. 80.6% of the predicted value; *p* = 0.018). There was a moderate negative correlation between the overall CT score for the extent of lung involvement and the TLC (r_s_ = −0.49; *p* = 0.003), as shown in Figure 2. The DL_CO_ did not differ statistically between the patients with and without signs of fibrosis, and there was no correlation between such signs and the CT score for the extent of lung involvement (r_s_ = −0.12; *p* = 0.504).

**Table 4:** Comparison between CT findings and PFT parameters in patients with respiratory symptoms at 12 months after COVID-19 (N = 37).

Parameter	Fibrosis like findings		Difference % (95% CI)	*P*
	Yes	No		
TLC (% predicted)*	68.6 ± 14.3	80.6 ±14.4	-12.1 (-21.9 to -2.2)	0.018
TLC < LLN*	12 (80.0)	11 (55.0)		0.237
DL_CO_ (% predicted)	68.8 ± 21.5	71.7 ± 20.1	2.9 (-17.0 to 11.2)	0.790
DL_CO_ < LLN	12 (75.0)	11 (55.0)		0.372

Data presented as n (%) or mean ± SD.

* Data available for only 36 of the 37 patients in the sample.

## DISCUSSION

Of the patients in our sample, 78.3% had at least one abnormality on chest CT. Fibrosis-like findings were present on the chest CT scans of 43.2% of the patients with respiratory symptoms related to post-COVID-19 condition at 12 months of follow-up. We also found that the TLC was significantly lower in the patients with signs of fibrosis on CT than in those without. A moderate negative correlation existed between the overall CT score for the extent of lung involvement and the TLC.

Pulmonary fibrosis is a known potential long-term complication of COVID-19^(18)^. Various studies have addressed the mechanisms leading to pulmonary fibrosis, which typically occurs after severe respiratory inflammation and injury^(18^–^20)^. In the lung, there can also be fibroproliferative damage, together with endothelial damage and angiogenesis^(18)^. Dyspnea, in the absence of a pulmonary lesion, could be related to inappropriate ventilation regulation resulting from dysautonomia, muscle deconditioning, or exercise-induced hyperventilation^(18^,^21^,^22)^.

The overall prevalence of any residual lung abnormalities in our study (78.3%) was higher than the pooled prevalence rate of 43.5% found in a previous large meta-analysis^(23)^. In addition, the proportion of patients in our sample with fibrosis-like findings on chest CT at 12 months of follow-up (43.2%) was higher than the 7.5% pooled prevalence reported in that same meta-analysis^(23)^, as well as being higher than the pooled prevalence of 20.6% reported in another large meta-analysis^(24)^. The higher prevalence of fibrosis-like findings on chest CT in our study may be due to the focus on patients with respiratory symptoms and because we included only patients who had been hospitalized for severe COVID-19. In contrast, other studies have included symptomatic and asymptomatic patients and may have included patients with less severe presentations of COVID-19. This hypothesis is strengthened by data from another study that demonstrated that signs of fibrosis at the 3-year follow-up visit were associated with a higher risk of residual respiratory symptoms^(25)^.

Several studies have shown that patients who recover from COVID-19 can later develop reduced DL_CO_ and evidence of a restrictive pattern of lung function^(25)^. In the present study, we did not find a significant association between signs of fibrosis and DL_CO_, as was reported previously^(26)^. However, another study demonstrated that the prevalence of reduced DL_CO_ at 24 months of follow-up was higher among the participants with fibrosis-like findings on chest CT than among those without such findings^(27)^. A previous study, in which patients were followed for 18–24 months after COVID-19^(28)^, showed lower TLC in patients with fibrosis-like findings on chest CT, similar to our finding. Nevertheless, our finding of a negative correlation between the overall CT score for the extent of lung involvement and the TLC differ from that of a smaller study (with only 26 patients), in which no correlation was found between the overall CT residual extent score at one year and the TLC^(29)^.

Our study has some limitations. First, not all the symptomatic patients in our cohort were included in our study sample, which reduced the size and statistical power of our sample. In addition, this was a single-center study conducted at a tertiary care hospital, which could create potential biases and confounding factors. Furthermore, we identified fibrosis solely on the basis of CT abnormalities, without histological correlation.

## CONCLUSION

In summary, we have assessed the prevalence of various chest CT findings in patients with respiratory symptoms attributed to post-COVID-19 condition, an understudied group. We have also shown that fibrosis-like findings and extensive lung involvement on follow-up chest CT are associated with lower TLC. It is noteworthy that a substantial number of symptomatic patients showed no abnormalities on chest CT, which highlights the complex mechanisms of injury and recovery in post-COVID-19 condition.

## Data Availability

Datasets related to this article will be available upon request to the corresponding author.
